# When an Event Is Perceived Depends on Where We Attend

**DOI:** 10.1177/2041669519858096

**Published:** 2019-06-19

**Authors:** Ljubica Jovanovic, Pascal Mamassian

**Affiliations:** Laboratoire des Systèmes Perceptifs, Département d’Études Cognitives, École Normale Supérieure, PSL University, CNRS, Paris, France; Neuropsychologie Cognitive, Physiopathologie de la Schizophrénie, Inserm UR 1114, University of Strasbourg, France; Laboratoire des Systèmes Perceptifs, Département d’Études Cognitives, École Normale Supérieure, PSL University, CNRS, Paris, France

**Keywords:** time perception, attention, temporal processing, spatiotemporal factors

## Abstract

Does the moment when an event is perceived depends on where it is presented? To measure when participants perceived events, they were first familiarized with trial duration, by watching the hand of a clock rotating. Then, the hand was removed, and stimuli were presented at a random time from the trial onset. Participants indicated the location where the hand would have been when the stimulus was presented. The stimuli’s eccentricity, the appearance, and location of the spatial features of the clock were varied. The targets were reported earlier if they were presented in spatial proximity to the clock outline, even when it was not presented during the trial. The effect was replicated with stimuli presented at the same distance from fixation but at different distances from the spatial features. In summary, the time of an event is perceived earlier if it is presented near attended features in the visual scene.

## Introduction

Directing attention to relevant locations in space improves the performance in a multitude of tasks (e.g., Cameron, Tai, & Carrasco, 2002; Carrasco & Yeshurun, 2009). There is, however, a complex relationship between attention and temporal processing.

For example, attended events are perceived earlier than not attended ones, as described in Titchener’s law of prior entry (Spence & Parise, 2010; Titchener, 1908). It is assumed that attention affects prioritization of the stimuli processing, resulting in the earlier apparent time of attended stimuli (Shore, Spence, & Klein, 2001). Furthermore, both sustained and transient attention can impair temporal resolution. Discrimination thresholds for two successive visual pulses are higher when transient attention is directed to their spatial location (Yeshurun & Levy, 2003), or when the attentional focus is diffused across a large area (Poggel, Treutwein, Calmanti, & Strasburger, 2006). Furthermore, when attention is divided across different, spatially disparate durations, spatial uncertainty decreases the precision of duration judgments (Ayhan, Revina, Bruno, & Johnston, 2012). Finally, when attention is divided between the temporal properties of a stimulus and its other features, the perceived duration of the stimulus is reduced, suggesting that the processing of duration shares the same, limited attentional resources as the processing of other features of that stimulus (Block, Hancock, & Zakay, 2010; Coull, Vidal, Nazarian, & Macar, 2004; Klapproth, 2011; Macar, Grondin, & Casini, 1994; Tse, Intriligator, Rivest, & Cavanagh, 2004; Zakay & Block, 2004).

Here, we report that when an event is perceived depends on where it is presented relative to some attended spatial location. In our task, participants estimated the passage of time, while simultaneously monitoring locations in the visual field where the stimulus appeared. At the beginning of the experiment, participants were familiarized with a fixed duration. For this purpose, they watched the hand of a clock rotating at a constant velocity to complete a single revolution. In the main part of the experiment, the hand of the clock was removed, and participants were asked to attend to an event that would occur within an interval that lasted the familiarized duration. The event of interest consisted of a pair of white discs that were flashed briefly at different locations on the horizontal meridian. At the end of the trial, participants reported their estimated time of the event by moving the cursor to indicate where the hand of the clock would have been at the time of the flash.

To investigate whether the reported time of visual events depended on the position of the event relative to the salient spatial features of the clock, we varied the appearance of the spatial features. We varied the size of the clock hand, so that the tip of the hand was in spatial proximity of different tested locations. Furthermore, the spatial features presented during the trial were different in the three experiments. In Experiment 1A, the outline of the clock and the stimulus outline were presented during the trial. In Experiment 1B, they were not presented, and only the fixation point was presented for the full duration of the trial. Finally, to disentangle the effects of the eccentricity (Carrasco, McElree, Denisova, & Giordano, 2003) and distance of the events from the salient features, in Experiment 2, we presented stimuli at the same eccentricity and varied their distance from the spatial features.

## Experiment 1

### Methods

#### Stimuli and apparatus

Stimuli were white discs with a radius of two degrees of visual angle (dva), flashed briefly (33 ms) at different positions on the screen. The background was mid-gray, and the fixation point was a white disc, size 1 dva, that changed luminance to dark gray as a preparation signal, just before the beginning of the trial. The hand of the clock had radius of 5 dva (Experiment 1A) or 9 dva (Experiment 1B), and it was also white. In Experiment 1A, a white circle, representing the face of the clock was presented during the familiarization phase and remained on the screen throughout the experiment. Each trial started and ended with a 33 ms pure tone, frequency 1 kHz. The experiment was conducted in a dark room.

Experiments were created using Matlab R 2016a and Psychtoolbox-3 (Brainard & Vision, 1997; Kleiner et al., 2007). Stimuli were presented on an LCD flat screen (ViewSonic V3F245), with diagonal 24 in., resolution 1,920 × 1,080 pixels, and refresh rate 60 Hz. The viewing distance was 30 cm.

The analysis of the data was conducted in the R Studio environment, using packages lme4 (Bates, Machler, Bolker, & Walker, 2014) and (Hothorn, Bretz, & Westfall, 2008) for mixed-effect regression analysis. We excluded trials with an error larger than 120 degrees from analyses (less than 5% of the trials were excluded).

#### Participants

Eleven participants took part in Experiments 1A and 1B. All but one of the participants (the first author, who took part in both experiments) were naive to the purpose of the experiment and gave written informed consent. The experiments were conducted in agreement with the Declaration of Helsinki and local ethics committee.

#### Procedure

At the beginning of the experiment, participants were familiarized with a fixed trial duration by watching the hand of a clock rotating at a constant speed, one cycle in 2 seconds. To provide an additional cue for remembering the duration of the trial, a brief tone (33 ms, 1 kHz) was presented at the beginning and the end of each revolution. During the main experiment, the hand of the clock was no longer presented, and participants were asked to fixate at the center of the screen during the trial. At the beginning and the end of the trial, two brief tones were presented. A stimulus was flashed briefly (33 ms) at a random time between the beginning and the end of the trial. To minimize attentional redirection to one hemifield if only one stimulus was flashed in periphery, two stimuli were simultaneously presented at the same eccentricity on either side of fixation. Participants were asked to attend to the time from the beginning of the trial, and estimate when the stimulus was presented within the time interval defined by the beginning and the end of the trial. When the trial ended, participants used the mouse to place a cursor at the position where the hand of the clock would have been at the time of the flash. On each trial, the timing of the target relative to the onset of the trial was chosen randomly. The stimulus was never presented 150 ms after the beginning or before the end of the trial. In five blocks, stimuli were presented at different locations in the visual field.

**Figure 1.**
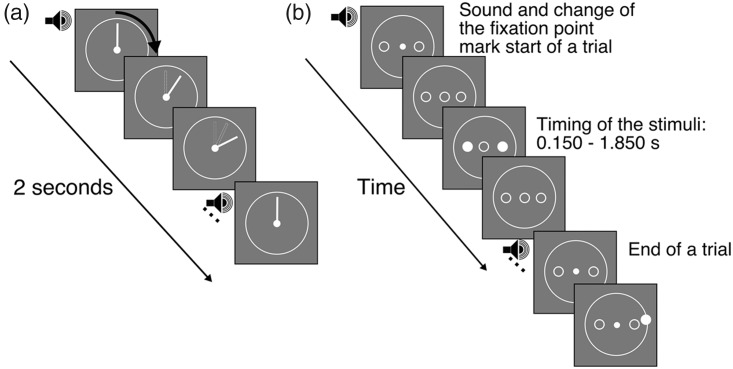
Representation of the temporal sequences of the familiarization phase and an experimental trial. (a) Familiarization with trial duration. At the beginning of the experiments, participants were presented with a clock. The hand of the clock rotated at a constant velocity, 2 seconds per revolution. A brief 1 kHz pure tone was presented at the beginning and at the end of each revolution, as an additional cue to facilitate learning of the trial duration. (b) Illustration of the stimulus sequence in Experiment 1. During the experiment, the hand of the clock was removed and the white circle representing the face of the clock remained on the screen. At the start of the trial, the fixation disc changed to a placeholder for the stimulus, and a brief tone was presented. After a random delay, two stimuli at either side of fixation were simultaneously presented. The trial ended after 2 seconds. The end of the trial was marked by a change of the placeholder to the fixation disc and a brief tone. Participants moved the mouse cursor to place it on the clock face at the location where the hand of the clock would have been at the time of the targets presentation. The procedure was similar in Experiment 1B, but the spatial features of the clock were reduced, and the response probe was changed. For details, see Figure 2.

**Figure 2.**
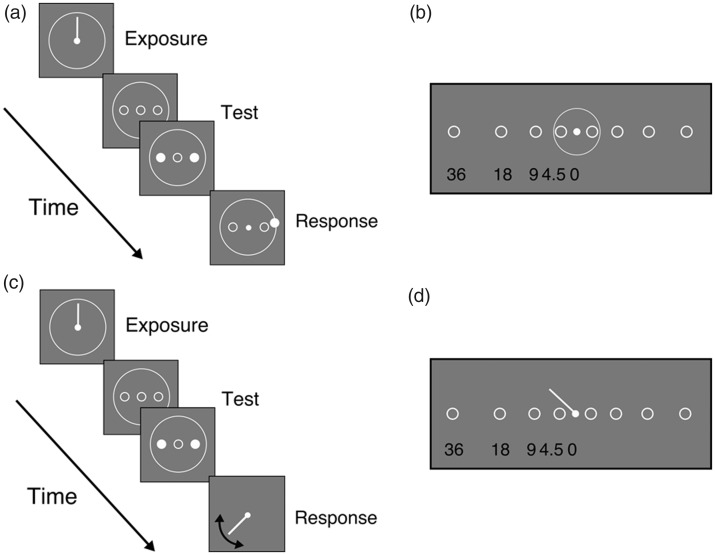
Illustration of layout and stimuli locations in Experiments 1A and 1B. (a) In Experiment 1A, during the exposure phase, the hand of the clock and the clock face were presented. In the test phase, the clock face and a placeholder for the stimuli were presented during the trial. Stimuli were briefly presented at the location of the placeholder, and participants estimated when the stimuli were presented. The response was given by placing the cursor on the clock face, at the location where the hand of the clock would have been at the time the stimulus was flashed. (b) Stimuli locations in Experiment 1A for the four tested eccentricities (4.5–36 dva) and their spatial relation to the face of the clock and the probe (c). In Experiment 1B, the spatial features of the clock were reduced, and the response probe was changed. In the exposure phase, only a rotating hand was presented. During the test phase, only the fixation point was presented, except when the stimuli were briefly shown. The response was given by rotating the hand of the clock, thereby indicating the time that elapsed from the beginning of the trial until the target was shown. The hand length was 9 dva (instead of 5 dva in Experiment 1A). (d) The four tested eccentricities (4.5–36 dva) and their spatial relation to the probe in Experiment 1B.

In Experiment 1A, the length of the hand of the clock was 5 dva. We tested five logarithmically equally spaced positions in the visual field, from 0 to 36 dva. During the exposure phase and the trial, the outline of the clock and stimuli were presented (Figure 1(a) and Figure 2(a) and (b)). In Experiment 1B, the length of the hand was 9 dva, and stimuli were presented at the same five eccentricities. We also minimized the spatial features by removing the clock outline and stimulus placeholders both from the exposure and the test phase (Figure 2(c) and (d)). The response probe was also changed: instead of placing the cursor on the outline of the clock, the hand of the clock reappeared and participants adjusted its orientation to match the perceived time, by moving the mouse. An illustration of the stimuli in the two experimental conditions is shown in Figure 2. Importantly, there was no uncertainty about the spatial locations of the stimuli, as the location of the stimuli was always the same within one block. In addition, the location of the features of the clock was not predictive of the stimulus location.

Before the start of the experiment and after each break, participants were presented with a full cycle of the rotating clock’s hand 15 times to help them memorize the duration of the trial, followed by a short training session with feedback. During the training, the target was always presented centrally. In the main part of the experiment, no feedback was provided. In Experiment 1A, participants completed 50 trials for each position of the stimulus (250 trials in total), and in Experiment 1B, 40 trials in each condition (200 trials in total). Perceived time of the stimuli at different positions was tested in separate blocks. After each block, participants had a short break, followed by a retraining. Each experiment was conducted in a single session, and each lasted for approximately 1 hour.

### Results

**Figure 3.**
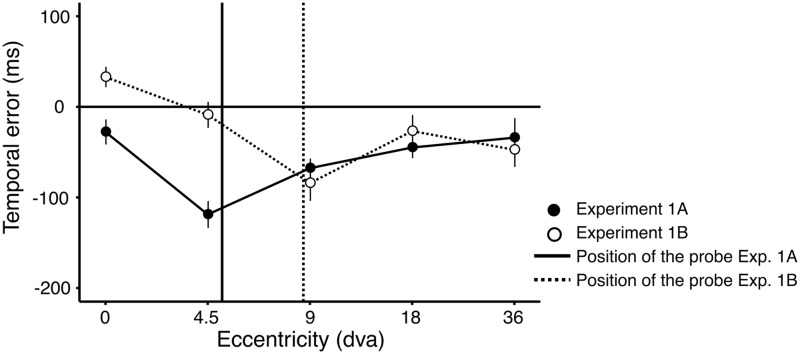
Results of Experiments 1A and 1B. The mean temporal errors in Experiments 1A and 1B are plotted against the tested locations. Temporal errors in Experiments 1A and 1B are shown in filled and open symbols, respectively. Negative errors indicate that the target was reported earlier than presented. Spatial location of probes in Experiments 1A and 1B is shown by the two dashed vertical lines. The targets are reported earlier if they are presented close to the spatial features of the clock (blue vertical dashed line for Experiment 1A and black for Experiment 1B). In addition, the targets are reported earlier if they are presented further in the periphery. Error bars represent standard error of the mean between participants (Loftus & Masson, 1994).

To quantify the performance, we calculated the temporal error as a difference between reported and presented time of the stimuli. Average temporal errors for the two experiments are shown in Figure 3. The performance in Experiments 1A and 1B is shown with filled and open symbols, respectively. The location of the spatial features in the two experiments is shown with two vertical lines. We found an overall bias to report events earlier than they were presented, indicated by negative errors. The bias was largest for events presented close to the spatial features of the display (the two vertical lines). In addition, there was an effect of eccentricity, and targets presented further in the periphery were reported earlier. We quantified the effect in the two experiments by means of two linear mixed-effect models. The temporal error was the dependent variable, and the tested locations in the visual field were included as a fixed factor. We also included participants as a random effect (intercept only), to account for additional variability. We tested whether the temporal errors were different for each eccentricity level, relative to the centrally presented stimuli, and corrected the significance level for simultaneous inference, using Bonferroni correction (Hothorn et al., 2008).

In Experiment 1A, we found an effect of eccentricity, *F*(4, 1411) = 10.704, *p* < .01. The contrasts revealed that the targets presented at all tested locations in the visual field were reported earlier than the targets presented at the central fixation. In Experiment 1B, the effect of eccentricity was also significant, *F*(4, 1411) = 8.53, *p* < .01. The targets presented at each peripheral location were reported earlier than the targets presented at the center of the visual field. In addition, the bias for the targets that were presented close to the spatial features of the display (4.5 dva) was greater than bias at other peripheral locations in Experiment 1A. In Experiment 1B, the bias for the target presented at 9 dva was greater for all the positions except for the bias for the targets presented at 36 dva.

## Experiment 2

In Experiments 1A and 1B, we tested when a visual event is perceived as a function of its location across the visual field. We found a large bias to report events earlier when they were presented near salient spatial features of the display. The spatial position of the stimuli reported with the greatest bias was different in the two experiments, suggesting that this effect is independent of the eccentricity-induced bias.

**Figure 4.**
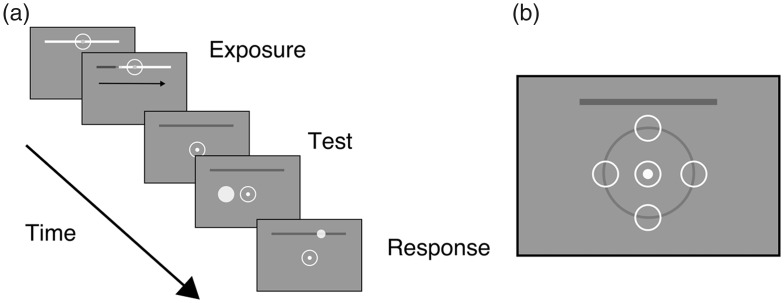
Illustration of the temporal sequence in Experiment 2. (a) At the beginning of the experiment, participants were presented with a horizontal bar. The bar changed its color gradually, indicating the passage of time. Brief tones were presented at the beginning and the end of each trial. (b) Illustration of the positions of stimuli in Experiment 2. We tested performance for five different positions of the stimuli: above, below, left, right of the fixation point, and at the location of the fixation. The horizontal gray bar was presented during the trial. The four locations around the fixation had the same eccentricity but were at different distances away from the probe.

To directly test this hypothesis, we conducted Experiment 2. Here, we presented targets on a virtual circle around the fixation, keeping the eccentricity constant across the tested positions (2 dva, Figure 4(b)). Instead of attending to the passage of time by tracking a rotating hand of the clock, participants were presented with a horizontal line at the top of the screen. In the exposure phase, the line slowly changed its color (from left to right) indicating the passage of time. As a result, stimuli presented below and above the fixation point could be presented at the same eccentricity, but with a different distance relative to the probe. If the distance from the probe was responsible for the underestimation of the presented time of the stimulus, we would expect that stimuli presented above the fixation are reported earlier than the stimuli below.

### Methods

#### Stimuli and apparatus

The passage of time was indicated by a change of color of a horizontal bar (size 8 dva) presented 6.5 dva above the fixation. To indicate the trial duration (2 seconds), the color of the bar gradually changed from white to gray (from left to right, see Figure 4(a)). During the main part of the experiment, the gray line remained on the screen. A pure tone duration 33 ms and frequency 1 kHz was presented at the start and end of each trial. Stimuli were white discs, presented on a Triton CRT screen (21 in.), with resolution 1,600 × 1,200 pixels and refresh rate 120 Hz. The viewing distance was 64 cm.

#### Participants

Six participants took part in the experiment. All but one of the participants (the first author) were naive to the purpose of the experiment and gave written informed consent. The experiment was conducted in agreement with the Declaration of Helsinki and local ethics committee.

#### Procedure

At the beginning of the experiment, participants were familiarized with a fixed trial duration by watching the line filling in, from left to right, with a constant speed. We presented a tone (33 ms, 1 kHz) at the beginning and the end of each trial, to provide an additional cue for remembering the trial duration. During the exposure phase, fixation was at the center of the probe. In the main part of the experiment, the gray line was presented, but it provided no temporal cues. Participants were asked to fixate at the center of the screen, and a single stimulus was flashed briefly (33 ms). The stimulus was presented at a random time relative to the onset of the trial. As in Experiments 1A and 1B, participants were asked to attend to the time from the beginning of the trial, and estimate when the stimulus was presented. When the trial ended, participants gave their answer by placing the cursor of the mouse on the horizontal line, to indicate the position they believed reflected the time of the flash. In five different blocks, we varied the position of the stimulus. The stimulus was presented 2 dva above, below, left or right from the fixation point, or at the location of the fixation (Figure 4(b)).

Before the experiment and after each break, participants had 15 practice trials with feedback. In each block, participants completed 50 trials (250 trials in total). The experimental session lasted approximately 1 hour.

#### Results

**Figure 5.**
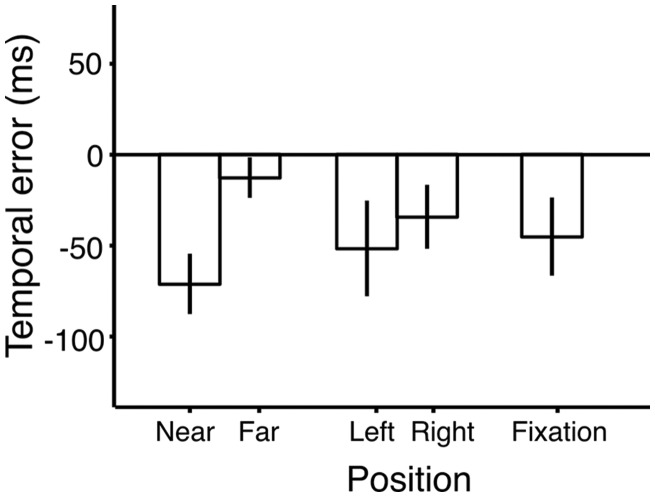
Results of Experiment 2. The mean temporal errors are plotted against the tested locations. There was an overall bias to report targets earlier, as indicated by negative errors. The targets are reported earlier if they are presented closer to the probe (above the fixation), relative to targets presented further from the probe (below fixation). Error bars represent standard error of the mean between participants (Loftus & Masson, 1994).

Mean temporal error across participants for the five tested target positions is shown in Figure 5. There was an overall bias to report targets earlier, indicated by average negative temporal errors. Importantly, targets were reported later if they were presented further away from the probe.

To test whether temporal errors were significantly different for different positions of the target relative to the probe, we conducted a linear mixed-effect model analysis. Temporal errors were the dependent, and positions on the screen were the predictor variable (categorical variable with five levels). We also included subject as a random effect to account for interindividual variability. There was a significant effect of the target position on temporal errors, *F*(4, 1487) = 4.05, *p* < .01. The contrasts revealed that the temporal error for targets presented at all positions except at the fixation were different from that of the target presented near the probe (i.e., above the fixation; Bonferroni corrected).

## Discussion

In the work reported here, we asked when an event is perceived when it is presented at different locations in the visual field. We adapted the procedure previously used to investigate the perceived time of decisions (Libet, Wright, & Gleason, 1983). In Experiments 1A and 1B, we varied the spatial features of the clock used to indicate when events were perceived (the size of the hand of the clock and the presence of the clock and stimulus outline). We found that the targets were reported earlier if they were presented in spatial proximity to the salient spatial features. The bias was found in both experiments; even though in Experiment 1B, no spatial features were presented during the trial. Importantly, the position in the visual field where the effect was found depended on the position of the spatial features.

The findings in Experiment 1 suggest that in order to perform the task, participants mentally attended to the positions of the clock’s hand during the trial. During this tracking, attention could be allocated to the spatial locations of the hand of the clock in the exposure phase. There are different possible interpretations of the observed bias. First, events that were presented close to the attended location could be perceived earlier. This hypothesis is in an agreement with previous work showing that attended targets are perceived earlier (Spence & Parise, 2010), and that attention can speed up processing at attended locations in space (Carrasco et al., 2003; Carrasco, Giordano, & McElree, 2006). Nevertheless, unlike in this previous work, here the location of the attended spatial features was not predictive of the stimulus location. In addition, the prior entry effect is considerably smaller when the attention is endogenously oriented to a target (Shore et al., 2001). Alternatively, adaptation studies provide evidence for the existence of local clocks across the visual field, whose speeds can be selectively changed by adaptation (Johnston, Arnold, & Nishida, 2006). In agreement with this hypothesis, targets presented at different eccentricities are processed by these localized mechanisms. Sharing attention at a particular spatial location, by allocating the central time-keeping mechanism that is shared across the eccentricities and the local clock at the same location, could cause slowing down of the timing mechanism at that location (Zakay & Block, 2010).

In Experiment 1, we presented stimuli at different locations across the periphery. Previous work has shown that temporal processing is not homogeneous across the visual field (Aedo-Jury & Pins, 2010; Kliegl & Huckauf, 2014), and that speed of temporal processing increases as a function of eccentricity (Carrasco et al., 2003). In agreement with this work, we found that stimuli were reported earlier in the periphery relative to when they were presented centrally. To disentangle these known effects of eccentricity and the effect we report here specific to the attended spatial location, we conducted Experiment 2. In this second experiment, we presented stimuli at the same eccentricity, but at different distances away from the assumed attended position. As predicted, stimuli that were presented closer to the probe were reported earlier.

In summary, we found that the time of an event is perceived earlier if it is presented near an attended feature in the visual scene. These results are consistent with previous work indicating a limited attentional resource for time estimation (Ayhan et al., 2012; De Montalembert & Mamassian, 2012; Morgan, Giora, & Solomon, 2008; Van Rijn & Taatgen, 2008). Our findings support the hypothesis of a single shared resource that is employed to compare the perceived time of an event at one location and the time elapsed from the beginning of the trial. It is an open question whether this mechanism is part of the dedicated temporal processing mechanism, or it is operating at another, possibly higher, level of cognitive processing.

## References

[bibr1-2041669519858096] Aedo-Jury F. Pins D. (2010). Time compression increases with eccentricity: A magnocellular property. Neuroreport, 21, 84–89. 19884866 10.1097/WNR.0b013e3283308d57

[bibr2-2041669519858096] Ayhan I. Revina Y. Bruno A. Johnston A. (2012). Duration judgments over multiple elements. Frontiers in Psychology, 3, 459. 23162507 10.3389/fpsyg.2012.00459 PMC3498874

[bibr29-2041669519858096] Bates, D., Mächler, M., Bolker, B., & Walker, S. (2014). Fitting linear mixed-effects models using lme4. *arXiv preprint arXiv*:1406.5823.

[bibr3-2041669519858096] Block R. A. Hancock P. A. Zakay D. (2010). How cognitive load affects duration judgments: A meta-analytic review. Acta Psychologica, 134, 330–343. 20403583 10.1016/j.actpsy.2010.03.006

[bibr114-2041669519858096] Brainard, D. H., & Vision, S. (1997). The psychophysics toolbox. *Spatial Vision*, *10*, 433--436. 9176952

[bibr4-2041669519858096] Cameron E. L. Tai J. C. Carrasco M. (2002). Covert attention affects the psychometric function of contrast sensitivity. Vision Research, 42, 949–967. 11934448 10.1016/s0042-6989(02)00039-1

[bibr5-2041669519858096] Carrasco M. Giordano A. M. McElree B. (2006). Attention speeds processing across eccentricity: Feature and conjunction searches. Vision Research, 46, 2028–2040. 16481020 10.1016/j.visres.2005.12.015 PMC2871539

[bibr6-2041669519858096] Carrasco M. McElree B. Denisova K. Giordano A. M. (2003). Speed of visual processing increases with eccentricity. Nature Neuroscience, 6, 699–700. 12819786 10.1038/nn1079 PMC3077107

[bibr8-2041669519858096] Carrasco M. Yeshurun Y. (2009). Covert attention effects on spatial resolution. Progress in Brain Research, 176, 65–86. 19733750 10.1016/S0079-6123(09)17605-7

[bibr9-2041669519858096] Coull J. T. Vidal F. Nazarian B. Macar F. (2004). Functional anatomy of the attentional modulation of time estimation. Science, 303, 1506–1508. 15001776 10.1126/science.1091573

[bibr10-2041669519858096] De Montalembert M. Mamassian P. (2012). Processing temporal events simultaneously in healthy human adults and in hemi-neglect patients. Neuropsychologia, 50, 791–799. 22285796 10.1016/j.neuropsychologia.2012.01.013

[bibr21-2041669519858096] Hothorn, T., Bretz, F., & Westfall, P. (2008). Simultaneous inference in general parametric models. *Biometrical Journal*, *50*(3), 346–363. 10.1002/bimj.200810425 18481363

[bibr22-2041669519858096] Johnston, A., Arnold, D. H., & Nishida, S. (2006). Spatially localized distortions of event time. *Current Biology*, *16*(5), 472–479. 10.1016/j.cub.2006.01.032 16527741

[bibr23-2041669519858096] Kleiner, M., Brainard, D., Pelli, D., Ingling, A., Murray, R., & Broussard, C. (2007). What's new in Psychtoolbox-3. *Perception*, *36*(14), 1.

[bibr11-2041669519858096] Klapproth F. (2011). Temporal decision making in simultaneous timing. Frontiers in Integrative Neuroscience, 5, 71. 22065490 10.3389/fnint.2011.00071 PMC3203373

[bibr12-2041669519858096] Kliegl K. M. Huckauf A. (2014). Perceived duration decreases with increasing eccentricity. Acta Psychologica, 150, 136–145. 24880978 10.1016/j.actpsy.2014.05.007

[bibr24-2041669519858096] Libet, B., Wright, E. W., Jr., & Gleason, C. A. (1983). Preparation-or intention-to-act, in relation to pre-event potentials recorded at the vertex. *Electroencephalography and clinical Neurophysiology*, 56(4), 367–372. 10.1016/0013-4694(83)90262-6 6193950

[bibr25-2041669519858096] Loftus, G. R., & Masson, M. E. (1994). Using confidence intervals in within-subject designs. *Psychonomic Bulletin & Review*, *1*(4), 476–490. 10.3758/BF03210951 24203555

[bibr13-2041669519858096] Macar F. Grondin S. Casini L. (1994). Controlled attention sharing influences time estimation. Memory & Cognition, 22, 673–686. 7808276 10.3758/bf03209252

[bibr26-2041669519858096] Morgan, M. J., Giora, E., & Solomon, J. A. (2008). A single “stopwatch” for duration estimation, a single “ruler” for size. *Journal of Vision*, *8*(2), 14–14. 10.1167/8.2.14 PMC2737673 18318640

[bibr27-2041669519858096] Poggel, D. A., Treutwein, B., Calmanti, C., & Strasburger, H. (2006). Increasing the temporal g (r) ain: Double-pulse resolution is affected by the size of the attention focus. *Vision Research*, *46*(18), 2998–3008. 10.1016/j.visres.2006.03.018 16677680

[bibr14-2041669519858096] Shore D. I. Spence C. Klein R. M. (2001). Visual prior entry. Psychological Science, 12, 205–212. 11437302 10.1111/1467-9280.00337

[bibr15-2041669519858096] Spence C. Parise C. (2010). Prior-entry: A review. Consciousness and Cognition, 19, 365–379. 10.1016/j.concog.2009.12.001 20056554

[bibr16-2041669519858096] Titchener E. B. (1908). Lectures on the elementary psychology of feeling and attention. New York, NY: Macmillan.

[bibr17-2041669519858096] Tse P. U. Intriligator J. Rivest J. Cavanagh P. (2004). Attention and the subjective expansion of time. Perception & Psychophysics, 66, 1171–1189. 15751474 10.3758/bf03196844

[bibr18-2041669519858096] Van Rijn H. Taatgen N. A. (2008). Timing of multiple overlapping intervals: How many clocks do we have? Acta Psychologica, 129, 365–375. 18849020 10.1016/j.actpsy.2008.09.002

[bibr28-2041669519858096] Yeshurun, Y., & Levy, L. (2003). Transient spatial attention degrades temporal resolution. *Psychological Science*, *14*(3), 225–231. 10.1111/1467-9280.02436 12741745

[bibr19-2041669519858096] Zakay D. Block R. A. (2004). Prospective and retrospective duration judgments: an executive-control perspective. Acta Neurobiologiae Experimentalis, 64, 319–328. 15283475 10.55782/ane-2004-1516

